# Increased use of diagnostic CT imaging increases the detection of stage IA lung cancer: pathways and patient characteristics

**DOI:** 10.1186/s12885-022-09585-2

**Published:** 2022-04-27

**Authors:** Charlotte Hyldgaard, Christian Trolle, Stefan Markus Walbom Harders, Henriette Engberg, Torben Riis Rasmussen, Henrik Møller

**Affiliations:** 1 Diagnostic Centre, Silkeborg Regional Hospital, Falkevej 1-3, Silkeborg, Denmark; 2 Department of Radiology, Odense University Hospital, Odense, Denmark; 3 The Danish Clinical Quality Program and Clinical Registries (RKKP), Aarhus, Denmark; 4 Department of Respiratory Disease and Allergy, Aarhus University Hospital, Aarhus, Denmark; 5 Danish Centre for Clinical Health Services Research, Aalborg University, Aalborg, Denmark

**Keywords:** Lung cancer, CT imaging, Diagnostics

## Abstract

**Background:**

At Silkeborg Regional Hospital, Denmark, the number of stage IA lung cancer increased after implementation of increased use of CT investigations and a corresponding reduction in chest X-ray. The aim of the present study was to understand the changes in referral pathways, patient characteristics and imaging procedures behind the observed increase in early-stage lung cancer.

**Methods:**

The referral and imaging pathways for all patients diagnosed with lung cancer in 2013–2018 were described based on manually curated information from the electronic health care systems and staging information from the Danish Lung Cancer Registry. We compared the clinical characteristics of patients diagnosed in 2013–2015 and in 2016–2018 after implementation of a change in the use of low dose CT scan (LDCT). For patients diagnosed in 2016–2018, stage IA lung cancer were compared to higher stages using univariable logistic regression analysis.

**Results:**

Five hundred and forty-seven patients were diagnosed with lung cancer in 2013–2018. Stage IA constituted 13.8% (34/247) in 2013–2015, and 28.3% (85/300) in 2016–2018. Stage IA patients in 2016–2018 were characterised by more comorbidity, fewer packyears and tended to be older than patients with higher stages. In 2016–2018, the largest proportion of stage IA patients (55%) came from within-hospital referrals. The majority of these lung cancers were detected due to imaging procedures with other indications than suspicion of lung cancer.

The proportion of stage IA increased from 12% (12/99) to 36% (47/129) (*p* < 0.001) for hospital referrals and from 17% (22/129) to 23% (38/165) for GP referrals (*p* = 0.21). The imaging procedures contributing to the increase in stage IA was contrast enhanced CT (22%¸11/51), LDCT (35%; 18/51) and X-ray followed by LDCT (25%; 13/51).

**Conclusion:**

The increased access to LDCT for patients referred from general practice and the increased hospital requested CT activity resulted in an increase in the number of stage IA lung cancers. Incidental findings on imaging performed for diagnostic purposes unrelated to suspicion of lung cancer contributed a large proportion of the increase.

**Supplementary Information:**

The online version contains supplementary material available at 10.1186/s12885-022-09585-2.

## Introduction

CECT is the recommended diagnostic method when there is a clinical suspicion of lung cancer. An alternative, which is used in organised screening programs for lung cancer is CT without contrast enhancement, which has a sensitivity much higher than conventional chest X-ray [1, 2]. Two alternatives have been considered to achieve timely diagnosis: organised screening for lung cancer in a defined population at risk, or extended access to CT imaging at a low threshold of suspicion of lung cancer. The first alternative has been shown to reduce lung cancer mortality in the screened population, [3–5] but the clinical effectiveness of increased access to referral to CT examination is uncertain [6]. For both alternatives, overdiagnosis may be a concern [7].

From 2016, Silkeborg Regional Hospital in Denmark used low dose CT (LDCT) as an alternative to conventional chest X-ray upon direct referral from primary care or as a supplement to chest X-ray for patients with respiratory symptoms who did not fulfil criteria for direct referral to CECT on suspicion of lung cancer.

**Fig. 1:**
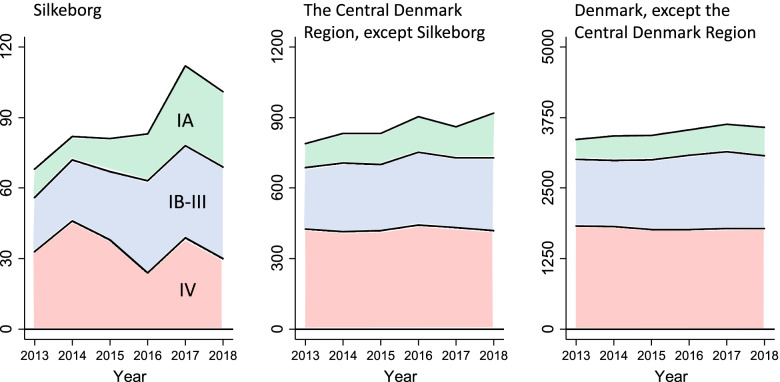
Distribution of early and higher stage lung cancers in 2013–2018 at Silkeborg Regional Hospital, the Central Denmark Region, except Silkeborg, and Denmark, except the Central Denmark Region

**Fig. 2:**
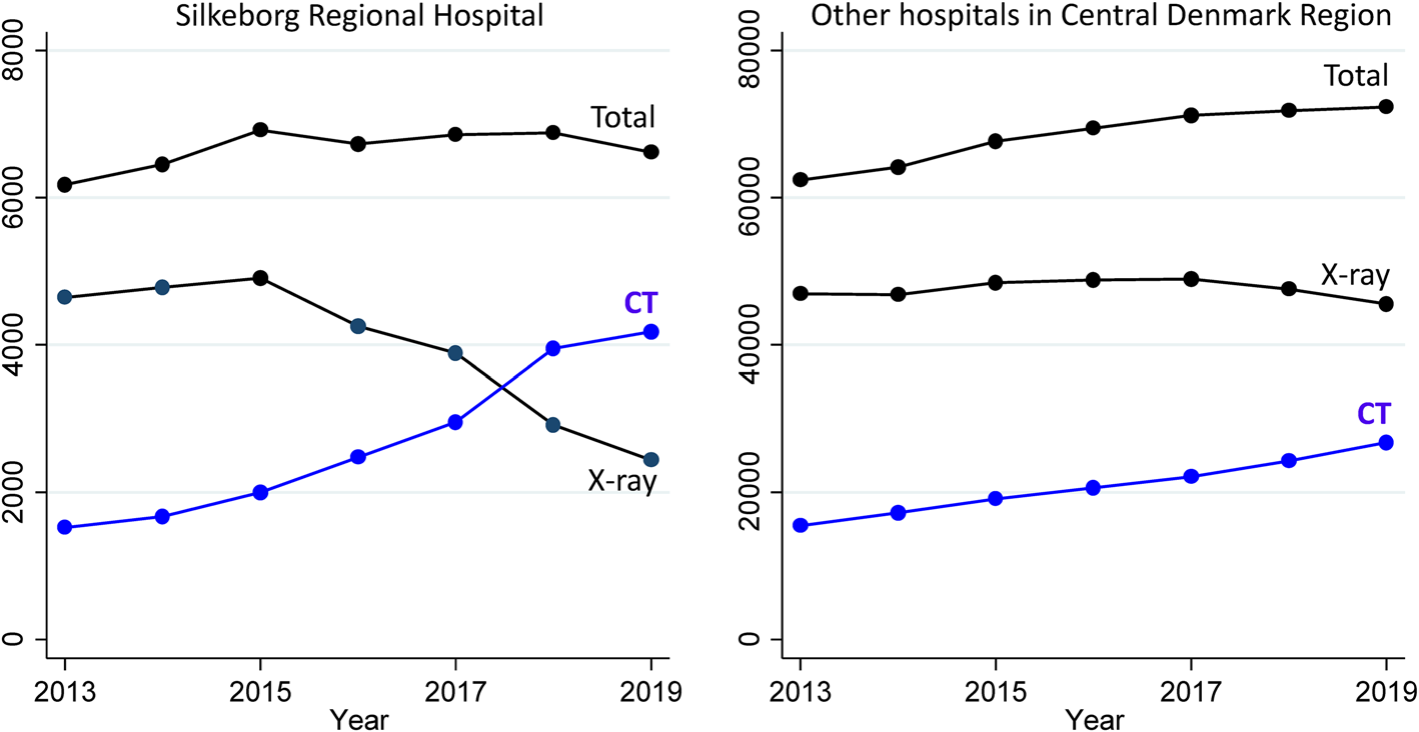
Thoracic imaging activity at Silkeborg Regional Hospital and at other hospitals in the Central Denmark Region in 2013–2019. Each individual contributes once to each X-ray and CT annual data point

The 2018 annual report from the Danish Lung Cancer Registry [8] showed an increase in the frequency of early stage lung cancers at Silkeborg Regional Hospital in 2016–2018 compared to previous years (Table 1 and Fig. 1). This small hospital diagnosed on average 101 lung cancers per year in 2016–2018 of which 38 (37.6%) were stage I. The increase in use of CT in 2016–2018 was approximately 2000 persons examined with CT each year, and about 40% of the volume of chest X-rays were replaced with CT examinations (Fig. 2).

**Table 1:** Frequencies of lung cancer in each stage-group, 2013-2015 and 2016-2018, with statistical comparison of the rate of change in each stage-group between geographical areas

	Silkeborg						The Central Denmark Region, except Silkeborg					Denmark, except the Central Denmark Region			
Clinical stage	Frequency 2013–2015	Frequency 2016–2018	Absolute change	Relative change	*p*-value vs. Region M.	*p*-value vs. Denmark	Frequency 2013–2015	Frequency 2016–2018	Absolute change	Relative change	*p*-value vs. Denmark	Frequency 2013–2015	Frequency 2016–2018	Absolute change	Relative change
IA	36	86	50	2.39	*0.004*	*0.001*	359	473	114	1.32	*0.23*	1205	1441	236	1.20
IB	13	29	16	2.23	*0.01*	*0.06*	182	171	-11	0.94	*0.07*	672	788	116	1.17
II	16	26	10	1.63	*0.26*	*0.43*	206	225	19	1.09	*0.39*	789	946	157	1.20
III	49	62	13	1.27	*0.69*	*0.34*	445	516	71	1.16	*0.18*	2125	2232	107	1.05
IV	117	93	-24	0.79	*0.09*	*0.14*	1262	1297	35	1.03	*0.32*	5415	5326	-89	0.98
NA	20	7	-13	0.35	*0.30*	*0.11*	190	116	-74	0.61	*0.14*	857	635	-222	0.74
Total	251	303	52	1.21	*0.15*	*0.06*	2644	2798	154	1.06	*0.33*	11,063	11,368	305	1.03

The present study was designed to infer the possible implications of this diagnostic intervention for early detection and diagnosis of lung cancer.

## Methods

### Data sources

We included all patients diagnosed with lung cancer between January 2013 and December 2018 at the Departments of Radiology or Internal Medicine at Silkeborg Regional Hospital, Denmark.

The Danish Lung Cancer Registry (DLCR) [9] was used to identify patients with a first diagnosis of lung cancer. Patients are included in the DLCR based on the first occurrence in the Danish National Patient Registry (DNPR) [10] of a diagnosis of cancer of the trachea or lung (ICD10 C33 and C34). Information of procedures and treatments in the DNPR are combined with information from the Danish Pathology Register [11] and the inclusion of incident cases in the DLCR is above 95% [12].

Patients from the hospital’s catchment area who did not have their primary diagnostic work-up at Silkeborg Regional Hospital were excluded.

Clinical data were obtained from the regional electronic clinical information system (Columna Clinical Information System (CCIS), Systematic), which contains all data regarding admissions and outpatient contacts at the hospitals in the region. Radiological referral and booking information were retrieved from four regional electronic archiving systems: Carestream RIS (Version 10.1.10), AGFA IMPAX 6.5.5.1608 "Enterprise unlimited", the CCIS imaging component, and the Regional Picture Archiving and Communication system. Radiology reports were retrieved manually from the radiology systems and regional electronic patient records.

#### Index image

We defined the index image as the first image with an abnormal finding leading to further investigation, e.g., an abnormal chest X-ray preceding a LDCT or CECT, was considered the index image. If a normal or non-suspicious chest X-ray preceded a LDCT or CECT with a suspicious finding, the CT was considered the index image. The index image was "None of the above" for CT scans with a clinical purpose other than lung cancer detection, e.g., cardiac CT, CT urography, or abdominal CT. When other imaging modalities showed signs of metastatic disease and raised the first suspicion of lung cancer, these were also included in the “None of the above” category, e.g., MRI of the brain or spine.

#### Clinical pathways

The examination date and type of all imaging procedures were registered until a CECT was performed, either isolated or as part of an ^18^FDG-PET/CT. The following clinical pathways were then assigned based on manual curation of all available data:

##### Lung cancer referral pathway

Direct CECT of the chest and upper abdomen on suspicion of lung cancer after GP referral, or referral from a hospital-based outpatient clinic or hospital ward. All CECTs were evaluated by a radiologist and reviewed in collaboration with a pulmonologist at multidisciplinary lung cancer team meetings.

##### Urgent referral pathway for non-specific serious symptoms

This pathway consisted of a standardised blood test panel, a chest X-ray and an abdominal ultrasound as the basic investigations. Patients received supplementary CECT of the chest, abdomen and pelvis when the radiologist considered it relevant or if the abdominal ultrasound provided insufficient information.

##### LDCT pathway

LDCT of the chest, which was not part of the above-mentioned cancer pathways. It included referrals directly from the GP and referrals from hospital. Furthermore, the pathway included patients above 40 years of age referred for an X-ray, who had a supplementary LDCT due to their smoking history (> 15 pack-years) if the x-ray was non-suspicious.

##### Not a defined clinical pathway

When the criteria for the three pathways mentioned above was not fulfilled.

After completion of the review of the clinical notes and establishment of the main imaging routes, we linked the clinical data to lung cancer stage, Charlson comorbidity index (CCI) derived from hospital discharge diagnoses at Danish hospitals in the 10 years before the lung cancer diagnosis, [13] and histology in the DLCR, using the Danish civil registration system [14].

Symptoms of lung cancer were gathered from referral information prior to diagnosis. “The red flag symptoms” cough, fatigue, dyspnoea, chest pain, weight loss, loss of appetite, abnormal spirometry, thrombocytosis, and haemoptysis were registered and classified according to Hamilton et al. [15]. Each patient was assigned the highest positive predictive value corresponding to either one symptom or a combination of two symptoms in accordance with the study by Hamilton et al.

#### Data analysis

The data for the present study include clinical and radiological characteristics, and the time interval from initiation of the diagnostic process to the diagnosis with lung cancer. The outcome variable was the clinical stage of the cancer. The change over time was predominantly in the frequency of stage IA cancers (Table 1) and we therefore did a univariable logistic regression analysis of the proportion of stage IA cancers out of all lung cancers.

Secondly, we used the entire dataset to give estimates in absolute numbers of the change in frequency of early-stage cancers that could be attributed to the explanatory variables. We used three-way tabulated data in the form of period*stage*covariate for these estimations, and Chi2-testing was used for comparison. The results were visualised as mosaic plots for selected variables.

The study was approved by the institutional review board at the Regional Hospital Central Jutland (Record number 1–45-70–37-20).

## Results

The starting point was 554 lung cancer patients identified in the DLCR as diagnosed at Silkeborg Regional Hospital. Seven patients did not have their primary diagnostic work-up at Silkeborg Regional Hospital and were excluded.

We observed 34 stage IA cancers (13.8%) in 2013–2015 and 85 stage IA cancers (28.3%) in 2016–2018, corresponding to an absolute increase of 51 stage IA cancers over time (Table 2). This increase was of the same magnitude as the overall increase from 247 to 300 cases. In the more advanced stage groups, overall numbers were constant but there was an increase in numbers of stage IB-III cancers and a decrease in stage IV cancers. The distribution of index images changed significantly with a decrease in chest x-ray from 60.3% to 37.0%, and increase in LDCT from 11.0% to 20.7% and CECT from 15.0% to 22.7% (Table 2, Index image). The use of the CECT imaging cascade increased from 11.3% to 19.7%; the use of the LDCT imaging cascade increased from 3.6% to 20.7%; while the use of X-ray and then CECT decreased from 52.2% to 26.0% (Table 2, Imaging Cascade). The use of the lung cancer referral pathway (direct CECT) increased from 6.5% to 8.0%; the use of LDCT pathway increased from 4.5% to 20.7%, and the use of the urgent referral pathway for patients with non-specific serious symptoms declined from 19.0% to 15.7% (Table 2, Clinical pathway).

**Table 2:** Description of 547 lung cancer patients, Silkeborg Regional Hospital 2013–2018, and comparison of distributions in two periods 2013-2015 (247)  and 2016-2018 (300)

	2013–2018 (547)		2013–2015 (247)		2016–2018 (300)	
	N	%	N	%	N	%
Outcome						
Clinical TNM stage						
IA	119	21.8	34	13.8	85	28.3
IB	42	7.7	13	5.3	29	9.7
II	42	7.7	16	6.5	26	8.7
IIIA	54	9.9	22	8.9	32	10.7
IIIB-IIIC	57	10.4	27	10.9	30	10.0
IV	208	38.0	116	47.0	92	30.7
NA	25	4.6	19	7.7	6	2.0
			*Chi2(6)* = *37.1; p* < *0.001*			
			*Chi2(5)* = *27.2; p* < *0.001 (ex. NA)*			
Person characteristics and constitution						
Age at diagnosis						
			*Median*		*Median*	
			*70.7*		*70.8*	
-59	66	12.1	27	10.9	39	13.0
60–69	192	35.1	89	36.0	103	34.3
70–79	216	39.5	97	39.3	119	39.7
≥ 80	73	13.3	34	13.8	39	13.0
			*Chi2(3)* = *0.66; p* = *0.88*			
Sex						
Male	293	53.6	132	53.4	161	53.7
Female	254	46.4	115	46.6	139	46.3
			*Chi2(1)* = *0.003; p* = *0.96*			
Charlson comorbidity score						
0	235	43.0	106	42.9	129	43.0
1	130	23.8	60	24.3	70	23.3
2	83	15.2	41	16.6	42	14.0
≥ 3	99	18.1	40	16.2	59	19.7
			*Chi2(3)* = *1.6; p* = *0.67*			
Pack-years						
≥ 40	252	46.1	116	47.0	136	45.3
20–39	187	34.2	78	31.6	109	36.3
10–19	41	7.5	18	7.3	23	7.7
0–9	43	7.9	24	9.7	19	6.3
NA	24	4.4	11	4.5	13	4.3
			*Chi2(4)* = *3.0; p* = *0.56*			
			*Chi2(3)* = *3.0; p* = *0.40 (ex. NA)*			
Morphology						
Small cell carcinoma	71	13.0	40	16.2	31	10.3
Adenocarcinoma	269	49.2	110	44.5	159	53.0
Squamous cell carcinoma	96	17.6	47	19.0	49	16.3
NSCLC unspecified	67	12.2	33	13.4	34	11.3
Other and NA	44	8.0	17	6.9	27	9.0
			*Chi2(4)* = *7.3; p* = *0.12*			
Referral						
Initiation of referral						
General practice	310	56.7	143	57.9	167	55.7
Hospital	237	43.3	104	42.1	133	44.3
			*Chi2(1)* = *0.27; p* = *0.60*			
Red flag symptoms (PPV, %)^a^						
0.0	169	30.9	70	28.3	99	33.0
0.1–0.9	247	45.2	109	44.1	138	46.0
≥ 1	131	23.9	68	27.5	63	21.0
			*Chi2(2)* = *3.5; p* = *0.18*			
Diagnostics						
Initial imaging conclusion						
Suspicious for cancer	414	75.7	194	78.5	220	73.3
Referral for follow-up	93	17.0	33	13.4	60	20.0
Not suspicious	40	7.3	20	8.1	20	6.7
			*Chi2(2)* = *4.4; p* = *0.11*			
Index image						
CECT	105	19.2	37	15.0	68	22.7
LDCT	73	13.3	11	4.5	62	20.7
Xray	260	47.5	149	60.3	111	37.0
None of the above	109	19.9	50	20.2	59	19.7
			*Chi2(3)* = *46.4; p* < *0.001*			
Imaging cascade						
CECT direct (includes 7 with PET)	87	15.9	28	11.3	59	19.7
LDCT or ULDCT	71	13.0	9	3.6	62	20.7
Xray then CECT	207	37.8	129	52.2	78	26.0
Xray then LDCT	82	15.0	33	13.4	49	16.3
Other	100	18.3	48	19.4	52	17.3
			*Chi2(4)* = *61.9; p* < *0.001*			
Clinical pathway						
Pathway						
Lung cancer referral pathway	40	7.3	16	6.5	24	8.0
Urgent referral pathway for non-specific serious symptoms	94	17.2	47	19.0	47	15.7
LDCT pathway	73	13.3	11	4.5	62	20.7
Not a defined clinical pathway	340	62.2	173	70.0	167	55.7
			*Chi2(3)* = *35.5; p* < *0.001*			
Timing of investigation and diagnosis						
Days from investigation to diagnosis						
Less than 31	431	78.8	205	83.0	226	75.3
31–60	33	6.0	12	4.9	21	7.0
61–179	29	5.3	9	3.6	20	6.7
≥ 180	53	9.7	20	8.1	33	11.0
NA	1	0.2	1	0.4	0	0.0
			*Chi2(4)* = *6.8; p* = *0.15*			
			*Chi2(3)* = *5.6; p* = *0.14 (ex. NA)*			

^a^Red flag symptoms: none, cough, fatigue, dyspnoea, chest pain, loss of weight, loss of appetite, abnormal spirometry, thrombocytosis, and haemoptysis

**Table 3:** Logistic regression analysis of 294 lung cancer patients, Silkeborg Regional Hospital 2016-2018. Outcome is cTNM stage IA

	cTNM					
	IA	Higher				
	(85)	(209)	%IA	OR	95% CI	
Patient characteristics and constitution						
Age at diagnosis						
	*Median*	*Median*				
	*72.0*	*69.9*				
-59	10	29	25.6	0.60	0.27	1.35
60–69	26	76	25.5	0.59	0.33	1.07
70–79	42	73	36.5	1.00		
≥ 80	7	31	18.4	0.39	0.16	0.97
				*Chi2(3)* = *5.9; p* = *0.11*		
Sex						
Male	40	120	25.0	1.00		
Female	45	89	33.6	1.52	0.91	2.52
				*Chi2(1)* = *2.6; p* = *0.11*		
Charlson comorbidity score						
0	29	99	22.7	1.00		
1	21	47	30.9	1.53	0.79	2.95
2	19	22	46.3	2.95	1.41	6.18
≥ 3	16	41	28.1	1.33	0.65	2.71
				*Chi2(3)* = *8.3; p* = *0.04*		
Pack-years						
≥ 40	32	99	24.4	1.00		
20–39	33	76	30.3	1.34	0.76	2.38
10–19	9	14	39.1	1.99	0.79	5.03
0–9	10	8	55.6	3.87	1.41	10.63
NA	1	12	7.7	0.26	0.03	2.06
				*Chi2(3)* = *7.9; p* = *0.048 (ex. NA)*		
Morphology						
Small cell carcinoma	2	29	6.5	0.12	0.03	0.52
Adenocarcinoma	57	99	36.5	1.00		
Squamous cell carcinoma	9	39	18.8	0.40	0.18	0.89
NSCLC unspecified	8	24	25.0	0.58	0.24	1.37
Other and NA	9	18	33.3	0.87	0.37	2.06
				*Chi2(4)* = *12.6; p* = *0.01*		
Referral						
Initiation of referral						
General practice	38	127	23.0	0.52	0.31	0.87
Hospital	47	82	36.4	1.00		
				*Chi2(1)* = *6.2; p* = *0.01*		
Red flag symptoms (PPV, %)*						
0.0	34	63	35.1	1.36	0.78	2.37
0.1–0.9	39	98	28.5	1.00		
≥ 1	12	48	20.0	0.63	0.30	1.31
				*Chi2(2)* = *4.0; p* = *0.13*		
Diagnostics						
Initial imaging conclusion						
Suspicious for cancer	40	176	18.5	1.00		
Referral for follow-up	36	24	60.0	6.60	3.55	12.27
Non-suspicious	9	9	50.0	4.40	1.64	11.79
				*Chi2(2)* = *38.9; p* < *0.001*		
Index image						
CECT	19	46	29.2	1.00		
LDCT	25	37	40.3	1.64	0.78	3.42
Xray	23	87	20.9	0.64	0.32	1.30
None of the above	18	39	31.6	1.12	0.52	2.42
				*Chi2(3)* = *7.4; p* = *0.06*		
Imaging cascade						
CECT direct	16	42	27.6	1.00		
LDCT or ULDCT	22	40	35.5	1.44	0.66	3.14
Xray then CECT	9	66	12.0	0.36	0.15	0.88
Xray then LDCT	21	28	42.9	1.97	0.88	4.41
Other	17	33	34.0	1.35	0.60	3.07
				*Chi2(4)* = *15.5; p* = *0.004*		
Clinical pathway						
Pathway						
Lung cancer referral pathway	5	19	20.8	1.00		
Urgent referral pathway for non-specific serious symptoms	12	33	26.7	1.38	0.42	4.52
LDCT pathway	25	37	40.3	2.57	0.85	7.78
Not a defined clinical pathway	43	120	26.4	1.36	0.48	3.87
				*Chi2(3)* = *5.2; p* = *0.16*		
Timing of investigation and diagnosis						
Days from investigation to diagnosis						
Less than 31	42	180	18.9	1.00		
31–60	8	11	42.1	3.12	1.18	8.23
61–179	12	8	60.0	6.43	2.47	16.72
≥ 180	23	10	69.7	9.86	4.36	22.27
				*Chi2(3)* = *41.1; p* < *0.001*		

Six patients with missing value for clinical stage are not included

*Red flag symptoms: none, cough, fatigue, dyspnoea, chest pain, loss of weight, loss of appetite, abnormal spirometry, thrombocytosis, and haemoptysis

### Stage IA vs. IB + comparison in 2016–2018

The age-distribution of stage IA patients was narrower than for more advanced cancers, and the stage IA proportion was highest (36.5%) in patients in their 70 s. The median age was higher in IA patients (72.0 years) than in other patients (69.9 years) (Table 3).

Stage IA patients had lower prevalence of smoking, with high odds-ratios for stage IA cancer in persons with less than 20 pack-years, and especially so with less than 10 pack-years (OR: 3.87; 95% CI: 1.41–10.63).

Stage IA patients had more comorbidity than patients with more advanced cancer (e.g., OR: 2.95; 95% CI: 1.41–6.18 in those with a comorbidity score of 2), and the proportion of stage IA cancers was lower in patients referred from their general practitioner than those referred from the hospital (OR: 0.52; 95% CI: 0.31–0.87).

The stage IA cancers were mostly adenocarcinoma: 57 cases, corresponding to 67% of all IA cancers.

The presence of red-flag symptoms [15] was negatively associated with stage IA cancer, with ORs of 1.36 (95% CI 0.78; 2.37); 1.00; and 0.63 (95% CI 0.30; 1.31) for PPV% groups 0; 0.1–0.9; and 1 + . The trend over the three red flag symptom groups was statistically significant: Chi2(1) = 4.0; *p* = 0.04 (data not shown).

In stage IA, the primary image was more often not-suspicious (chest x-ray) (OR: 4.40; 95% CI: 1.64–11.79) or requiring a follow-up investigation (CT detected nodules) (OR: 6.60; 95% CI: 3.55–12.27). The time from initial imaging to lung cancer diagnosis was higher in stage IA patients, e.g., more often exceeding six months (OR: 9.86; 95% CI: 4.36–22.27).

The detection and diagnosis of stage IA cancer was associated with the LDCT imaging cascade (OR: 1.44; 95% CI: 0.66–3.14), and especially so when an initial X-ray examination was followed by LDCT (Imaging cascade X-ray then LDCT: OR: 1.97; 95% CI: 0.88–4.41), both compared with the imaging cascade direct CECT. These results are consistent with the detection of stage IA cancer being highest in patients where LDCT and not CECT was the index image (OR: 1.64; 95% CI: 0.78–3.42) and in the LDCT pathway compared with the lung cancer referral pathway (OR: 2.57; 95% CI: 0.85–7.78), although not statistically significant. The detection of stage IA cancer in the LDCT imaging cascade was strongest in patients referred from their GP (OR: 4.03; 95% CI 1.21–13.42; data not shown).

All lung cancer patients at Silkeborg Regional Hospital underwent diagnostic follow up with PET-scan and biopsy as necessary according to current lung cancer guidelines. Almost all patients had a pathological tissue diagnosis. Of the 85 stage IA patients in the 2016–2018 cohort, 94% were offered radical treatment; surgical resection in 58 cases and primary radiotherapy in 22 cases. In the remaining five cases, no record of treatment was available.

### Contributions to the change in number of stage IA cancers

**Fig. 3:**
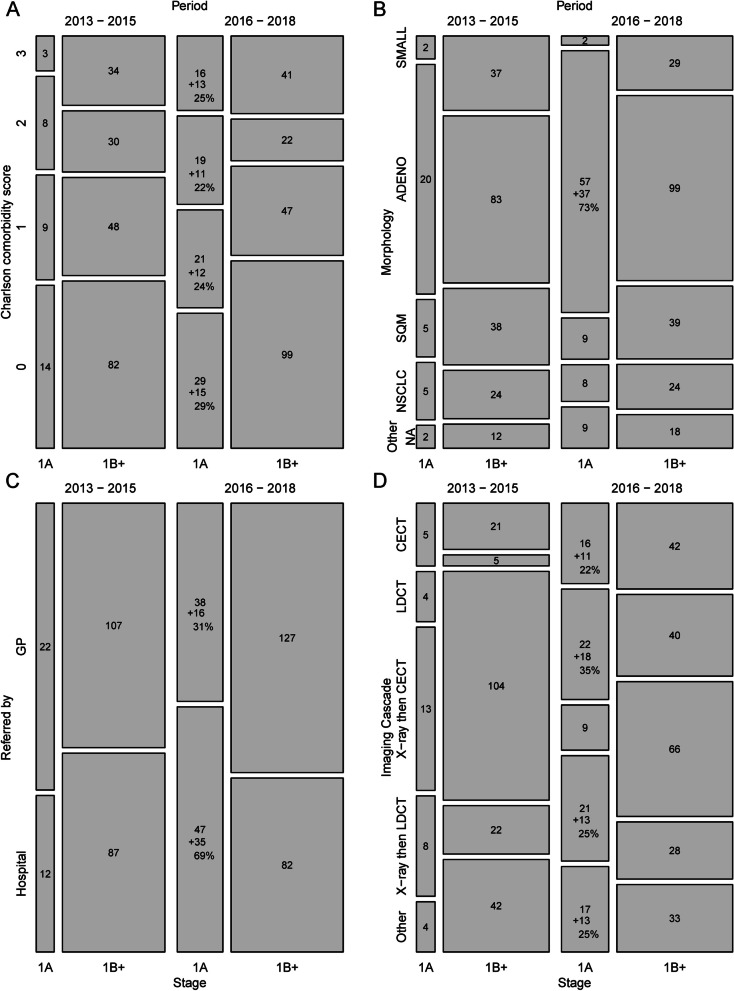
Mosaic plots of Charlson comorbidity score (**A**), morphology (**B**), initiation of referral (**C**) and imaging cascade (**D**). The area of each square is proportional to the number of persons in that subgroup. The numbers are the frequencies in each group. For Stage 1A cancers in 2016–2018 the change from 2013–2015 to 2016–2018 and the increase as a percentage of the overall increase are also shown

Figure 3 shows the absolute numbers of stage IA patients and higher tumor stage patients in the two time-periods 2013–2015 and 2016–2018, in subgroups defined by comorbidity (Fig. 3A), morphology (Fig. 3B), origin of referral (Fig. 3C), and imaging cascade (Fig. 3D).

For comorbidity, the proportion of patients with CCI ≥ 3 was higher in stage IA patients than in other patients in 2016–2018, and the increase in numbers of stage IA in these comorbid patients was 24 cases (24/51) corresponding to 47% of the overall increase of 51 stage IA patients.

For morphology, the increase in adenocarcinoma was 73% (37/51) of the total increase in stage IA cancers.

For origin of referral, stage IA patients in 2013–2015 were most often referred by their GP, but this changed in 2016–2018 where these patients were mostly referred from the hospital. The majority of the increase in the number of IA cancers (35 cases, (35/51) or 69% of the total increase) came from hospital referrals.

For imaging cascade, most of the imaging procedures contributed to the overall increase in IA numbers from 2013–2015 to 2016–2018: CECT (22%; 11/51), LDCT (35%; 18/51), X-ray followed by LDCT (25%; 13/51), and other imaging (25%; 13/51). Stage IA detected by initial X-ray and then CECT declined by 8% ( -4/51). Results for LDCT were similar to this in analyses of index image (39%) and clinical pathway (39%) (data not shown).

Stage IA patients had longer duration from first imaging to diagnosis, and more so in 2016–2018 where the increase in stage IA numbers came from patients where this duration exceeded one month (28 cases; (28/51), 55% of total increase), and especially where the duration exceeded six months (23 cases; (23/51), 45% of the total) (data not shown).

Age, morphology and follow-up time was comparable between GP- and hospital referred patients, but comorbidity measured by the CCI tended to be higher among hospital referred patients (p = 0.056) (data not shown).

Patients who were referred by their general practitioner and initially examined with LDCT (16 patients) had odds-ratio of 0.73 (0.37–1.46) for stage IA cancer, compared with those referred from the hospital (47 patients). The highest proportion of IA cancer was in patients referred by the general practitioner and examined first with X-ray and then with LDCT (14 patients) (OR: 1.53; 95% CI: 0.68–3.40) (data not shown).

The statistical analyses in Tables 2 and 3 are univariate analyses of the observed frequencies. We did extensive multivariate analyses to verify that the reported odds ratios in Table 3 were not confounded by other variables in the dataset. Examples of these analyses are shown in the Supplementary table 1 where all odds ratios are adjusted for age, sex and pack-years.

## Discussion

We identified a *priori* several possible and not mutually exclusive mechanisms that could contribute to the association between CT use in the hospital and the incidence of early-stage lung cancer. The following discussion is structured according to those possible mechanisms*.*

### The role of LDCT in the detection of stage IA lung cancer in 2016–2018

It is evident from these data that many diagnoses of early-stage lung cancer at Silkeborg Regional Hospital in 2016–2018 involved the use of LDCT in the diagnostic process. Of the 85 stage IA cases, 26% (22/85) involved the direct use of LDCT (Table 3, Imaging cascade), and contributed to 35% (18/51) of the increase in the number of stage IA cases from 2013–2015 to 2016–2018 (Fig. 3D). The supplementation with LDCT based on patient risk profile was unique to Silkeborg, but it is not known if a setting without access to LDCT would result in CECT or an X-ray follow-up potentially resulting in the same diagnosis. Overall, direct LDCT or LDCT following an X-ray was seen in 51% of patients with a stage IA cancer.

### The contribution of the general practitioner’s referral choice

The diagnostic centre at Silkeborg offered several referral options to the GPs in the area. This included the introduction of a referral route directly to LDCT aimed at *low risk but not no risk* patients [16] who were considered not to fulfil the criteria for the principal CECT referral for patients with symptoms of lung cancer, and in whom a referral to X-ray would be considered sub-optimal.

Patients who were referred by their general practitioner and diagnosed with stage IA cancer (38 patients) were initially examined with X-ray and LDCT in similar numbers (16 and 17, respectively). The GP referred patients with the highest yield of stage IA cancer was those initially investigated by X-ray and then by LDCT. This illustrates that the *low risk but not no risk* category is difficult to identify, even when a specific referral option exists for such patients. A controlled trial has earlier been reported from Denmark, where an option of direct GP referral to LDCT was compared with a standard scenario without this added option [17]. The study population yielded 331 incident cases of lung cancer but found no effect of the added LDCT option on the stage distribution. This may illustrate that stage IA lung cancer most often is non-symptomatic, which renders it likely that other mechanisms than GP referral choice led to the increase in stage IA cancer at Silkeborg Regional Hospital.

### The origin and contribution of incidental findings

This investigation started with the observation of a highly statistically significant increase in the incidence of stage IA cancers. This increase was much stronger for hospital referrals where the proportion of stage IA increased threefold from 12% (12/99) to 36% (47/129) (*p* < 0.001), than for GP referrals where the increase was 1.5-fold from 17% (22/129) to 23% (38/165) (*p* = 0.10).

In 2016–2018, the largest proportion of stage IA patients (55%) came from within-hospital referrals (Fig. 3) explaining 69% of the increase between the two periods. The majority of these lung cancers were detected due to imaging procedures with other indications. A wide range of imaging procedures contributed. The associations between comorbidity and stage IA and between hospital referral and stage IA both point strongly towards the contribution of incidental findings to the incidence of stage IA cancer.

### Evidence suggestive of possible overdiagnosis

Overdiagnosis in cancer is the detection of a tumour that would not otherwise have become clinically apparent in the life-time of the individual. Overdiagnosis is often an intrinsic feature of screening, which by its nature seeks to detect occult disease in asymptomatic individuals.

The tendency of a higher median age among patients with stage IA compared with higher stages may suggest an extent of overdiagnosis, although the difference is not statistically significant. The increase in the number of small, slow-growing tumours needing long follow-up before a diagnosis was made, may also point towards this. These characteristics (age, adenocarcinoma morphology and time-to-diagnosis) were similarly distributed in the hospital and GP referrals (data not shown).

There is substantial heterogeneity in growth rates of LDCT screening detected lung cancers, indicating that a reservoir of slowly or non-growing lung cancer exists. [18] LDCT scans have a much higher resolution than chest radiography, thus increasing its ability to detect the reservoir of indolent and slow-growing pathology.

The conclusive appraisal of overdiagnosis requires observation on the lung cancer mortality rate in the catchment area of Silkeborg Regional Hospital in future years.

### Practical implications of these results

A full evaluation of the Silkeborg protocol requires attention to the broad range of patients and outcomes of thousands of CT examinations, not just the lung cancers that were detected. The high use of CT may contribute to the management of a wide range of other conditions, but the benefits should be balanced with the possibility of incidental findings and overdiagnosis that may cause unnecessary or harmful interventions.

It is evident from these data that the practice change at Silkeborg Regional Hospital has had the effect of increasing the rate of detection of stage IA lung cancers, and that half of this detection has involved the use of LDCT imaging. The results show that a large proportion of the increase in these early-stage cancers are in the form of incidental findings. The incidental finding of serious disease may certainly be of benefit to the patient, indeed life-saving, but a clinical practice set up primarily to make incidental findings is similar to a screening programme, and its design and implementation should follow the principles of analysis of benefits and costs that apply to an organised screening programme.

With the emerging evidence for lung cancer screening, [3, 4] it is likely that focus will be on the implementation of a national screening programme rather than the use of LDCT as an alternative “third pathway” between chest x-ray and CECT.

A limitation of this study is that it had to be conducted as an observational epidemiological study, relying on data that could be retrieved by review of the clinical notes. The number of patients at Silkeborg Regional Hospital is small and similar data and analyses from other Danish hospitals are not available. An intervention and practice change on the scale of the transition from X-ray to CT imaging at Silkeborg Regional Hospital should preferably be designed with prospective collection of data on the indications and the results of the investigations, hereby making the evaluation a protocolled part of the intervention.

The present study reports the single-institution experience of increased detection of early-stage lung cancer upon a large increase in the use of thoracic CT examination of patients at the hospital. The results are likely to be generalisable to other hospitals with a similar high level of routine CT activity.

## Conclusion

The increased access to LDCT for patients referred from general practice and the increased hospital requested CT activity resulted in an increase in the number of stage IA lung cancers. Incidental findings on imaging performed for diagnostic purposes unrelated to suspicion of lung cancer contributed a large proportion of the increase.

## Supplementary Information

**Additional file 1: Supplementary table 1.**Logistic regression analysis of 294 lung cancer patients, Silkeborg Regional Hospital 2016-2018. Multivariate analysis example.

## Data Availability

The data that support the findings of this study are not publicly available and were used with permission from the Regional Hospital Central Jutland for the current study. Data are however available upon reasonable request and with permission of the Regional Hospital Central Jutland.
